# Network Analysis Reveals That Headache-Related, Psychological and Psycho–Physical Outcomes Represent Different Aspects in Women with Migraine

**DOI:** 10.3390/diagnostics12102318

**Published:** 2022-09-26

**Authors:** César Fernández-de-las-Peñas, Lidiane L. Florencio, Umut Varol, Juan A. Pareja, Carlos Ordás-Bandera, Juan A. Valera-Calero

**Affiliations:** 1 Department of Physical Therapy, Occupational Therapy, Rehabilitation and Physical Medicine, Universidad Rey Juan Carlos, 28922 Alcorcón, Spain; 2 VALTRADOFI Research Group, Department of Physiotherapy, Faculty of Health, Camilo Jose Cela University, 28692 Villafranca del Castillo, Spain; 3 Department of Neurology, Hospital Quirón Pozuelo, 28223 Madrid, Spain; 4 Department of Neurology, Hospital Rey Juan Carlos, 28933 Móstoles, Spain; 5 Department of Physiotherapy, Faculty of Health, Camilo José Cela University, 28692 Villafranca del Castillo, Spain

**Keywords:** migraine, pressure pain, disability, network analysis

## Abstract

Evidence supports that migraine is a complex pain condition with different underlying mechanisms. We aimed to quantify potential associations between demographic, migraine-related, and psychophysical and psychophysical variables in women with migraine. Demographic (age, height, and weight), migraine-related (intensity, frequency, and duration), related-disability (Migraine Disability Assessment Scale, Headache Disability Inventory), psychological (Hospital Anxiety and Depression Scale), and psycho–physical (pressure pain thresholds -PPTs-) variables were collected from a sample of 74 women suffering from migraine. We calculated adjusted correlations between the variables by using a network analysis. Additionally, we also calculated centrality indices to identify the connectivity among the variables within the network and the relevance of each variable in the network. Multiple positive correlations (ρ) between PPTs were observed ranging from 0.1654 (C5-C6 and tibialis anterior) to 0.40 (hand and temporalis muscle). The strongest associations within the network were those between migraine attack frequency and diagnosis of chronic migraine (ρ = 0.634) and between the HDI-E and HDI-P (ρ = 0.545). The node with the highest strength and betweenness centrality was PPT at the second metacarpal, whereas the node with the highest harmonic centrality was PPT at the tibialis anterior muscle. This is the first study applying a network analysis to understand the underlying mechanisms in migraine. The identified network revealed that a model where each subgroup of migraine-related, psychological, and psycho–physical variables showed no interaction between each variable. Current findings could have clinical implications for developing multimodal treatments targeting the identified mechanisms.

## 1. Introduction

Primary headaches are the pain disorders most commonly attended by neurologists in clinical practice and are ranked among the top ten disabling conditions worldwide in individuals under the age of 50 years [1,2]. The global prevalence of migraine is estimated to be up to 11.6% (13.8% in females, 6.9% in males) [3]. 

Current theories support the presence of complex mechanisms for underlying pathogenesis of migraine [4]. Although the main hypothesis associate migraine to an abnormal neuronal excitability with cortical spreading depression and to a hyperexcitability of trigeminovascular pathways [4], other factors such as pressure pain hyperalgesia [5], emotional–psychological factors [6], sleep disorders [7], or genetics [8] are also involved in migraine pathogenesis in a complex matrix. Previous studies investigating the potential associations between different aspects of migraine have commonly used Pearson Product-Moment Correlations or linear regressions. However, it should be noted that these analyses ignore the potential associations to arise from their interaction with another variable, as it is the case of Pearson Product-Moment Correlations, or ignore the possibility of bidirectional relationships between the variables, as it is the case of linear regression [9]. These potential limitations can be addressed using network analysis [10]. 

Network analysis provides a statistical method to identify the most important variables in the identified network, which could be used to potentially design better therapeutic strategies [11]. In fact, network analysis is starting to be used to better understand the complexity of chronic pain conditions such as tension-type headaches [12], fibromyalgia syndrome [13], carpal tunnel syndrome [14], or post-COVID pain [15]. From a network perspective, migraine represents a chronic pain condition sustained by multivariate interactions between migraine-related, emotional/psychological, and physiological systems. Since no previous study has used network analysis in individuals with migraine, we applied network analysis to better understand those potential interactions between migraine-related, emotional/psychological, and psychophysical variables. Therefore, the aim of this study was to describe a network including demographic, migraine-related, and emotional/psychophysical or psychophysical variables in women with migraine to illustrate its potential use for a better understanding of underlying mechanisms of migraine.

## 2. Methods

### 2.1. Participants

Consecutive women with migraine recruited from a headache unit located in a tertiary university-based hospital were screened for eligibility criteria between January 2017 and December 2018. Migraine diagnosis was conducted according to the third edition criteria of the International Classification of Headache Disorders (ICHD-III), the beta [16] or final [17] version by neurologists with more than 20 years of experience. Participants were excluded if they presented: 1, other concomitant headache; 2, neck or head trauma; 3, cervical herniated disk; 4, comorbid underlying medical disease; 5, fibromyalgia; 6, had changed medication intake within the previous 6 months; 7, had received any treatment including anesthetic blocks, botulinum toxin or physical therapy the previous 6 months; or, 8, pregnancy. The study was properly revised and approved by the Local Human Ethical Committee of Hospital Rey Juan Carlos (HRJ 07/14). All participants read and signed a written consent form prior to their participation in the study.

Participants were examined when they were headache-free or, in those with chronic migraine, when the intensity of the migraine attack the day of the evaluation was ≤3 points on the numerical pain rate scale (NPRS). Patients were asked to avoid taking any analgesic or muscle relaxant 24 h before their examination.

### 2.2. Migraine-Related Variables

Participants registered in a 4-week headache diary their number of days per week with a migraine attack, the duration of the attack (hours/day), and the pain intensity of each migraine attack on an 11-points NPRS (0: no pain; 10: the worst unimaginable pain) [18].

### 2.3. Disability-Related Variables

Since migraine is a highly disabling condition, we used two general questionnaires of headache and one specific for migraine.

The Headache Disability Inventory (HDI) is a 25-item questionnaire focusing on the impact of headache on emotional functioning and daily activities [19] with good test-retest reliability [20] and therefore was used for assessing the headache-related disability. Thirteen items evaluate the emotional burden (HDI-E, score from 0 to 52), and 12 items the physical burden (HDI-P, score from 0 to 48) of headache. A greater score suggests a greater headache-related burden. The Headache Impact Test (HIT-6) was the second headache-related general variables used for assessing a headache’s impact [21]. The final score varies between 36 and 78 and higher scores suggest a more severe impact. 

The Migraine Disability Assessment Scale (MIDAS) is a specific-disease questionnaire used to assess the degree of migraine-related disability in daily activities (work or school, family, and social) [22]. The MIDAS includes 5 questions related to days of partial or total loss within the last 3 months on: 1, paid work or school; 2, household chores; 3, family, social and leisure activities. The final score comes from the sum of the missed days regarding the 3 three activities. In the current study, we used the Spanish version of the MIDAS questionnaire which has proved to be a valid and reliable tool [23].

### 2.4. Emotional/Psychological Variables

For assessing the presence of anxiety and depression, we used The Hospital Anxiety and Depression Scale (HADS). This self-reported questionnaire consists of seven items assessing the presence or absence of anxiety (HADS-A) and seven analyzing depressive symptoms (HADS-D) [24]. Scores of each item range from 0 to 3 in a 4-point Likert scale (where higher scores indicate a worse health status), providing a maximum of 21 points for each subscale and 42 points in total. This scale was selected as it demonstrated good internal consistency in people with headaches [25].

### 2.5. Psycho–Physical Variables

An electronic pressure algometer (Somedic^®^ Algometer, Sollentuna, Sweden) was used to calculate pressure pain thresholds (PPTs). We assessed PPT bilaterally over the temporalis muscle (a trigeminal point), C5-C6 joint (an extra-trigeminal point), as well as second metacarpal and tibialis anterior muscles (two remote pain-free points), as these sites have been found to exhibit pressure pain hyperalgesia in migraine sufferers [26]. A training session for familiarization with the procedure was conducted. The mean of 3 trials on each point, with a 30s resting period, was calculated and used in the analysis. The order of assessment was randomized between points on each participant. Since no side-to-side differences were observed (Student’s *t*-test), the mean of both sides for each point was used in the network.

### 2.6. Statistical Analysis

#### 2.6.1. Software and Packages

Data analyses were conducted on R software v.4.1.1 for Windows 10. Several packages were used for different purposes: qgraph (v.1.6.9) and glasso (v.1.11) for network estimation, igraph (v.1.2.6) for community detection, huge (v.1.3.5) for variable transformation, bootnet (v.1.4.3) for stability analysis, CINNA (v.1.1.54) for harmonic centrality measurement and mgm (v. 1.2-12) for estimating k-order mixed graphical models [27,28,29].

#### 2.6.2. Exploratory Analysis

After conducting an exploratory data analysis on the dataset, 1 missing value was found for the MIDAS score. Since the removal of this missing value would result in loss of 1.35% of the data (73 records), this record was dropped from the dataset.

#### 2.6.3. Network Estimation

The procedure of network analyses has been extensively described in previous papers by our research group [12,13,14,15]. We will briefly describe some important points here. Networks represent the variables as nodes and the associations between the variables as edges. The current network included 16 nodes, 15 continuous variables (age, psycho–physical, psychological, headache and health related variables), and one categorical variable (type of migraine). 

We used the least absolute shrinkage and selection operator (LASSO, ℓ1-regularization) for including as few false positives as possible [27]. LASSO utilizes a tuning parameter λ (the strength of the penalty) for controlling the sparsity level that directly penalizes the likelihood function for the sum of absolute parameter values [27], and for creating a network structure that minimizes the number of spurious edges while maximizing the number of true edges [11]. 

#### 2.6.4. Node Centrality

Centrality indices provide an estimation of the relevance of each node in the network. We calculated strength, harmonic, and betweenness centrality [30,31,32,33].

Node strength considers the total involvement of a particular node in a network for identifying the most relevant node. The node with a higher strength centrality may involve changes to the other nodes [33]. However, strength centrality does not consider the mediating role of other nodes and the number of connections with other nodes. Accordingly, using other centrality indicators is important to derive accurate conclusions [34]. 

Harmonic centrality was calculated instead of closeness centrality for the unconnected nodes found in the current network [35]. A node with high harmonic centrality could be easily affected by changes in another node’s value directly or through changes in other nodes [33]. 

Finally, a node with a high betweenness centrality acts as an intermediary in the transmission of information or resources between other nodes or even clusters of nodes in the network [31]. 

#### 2.6.5. Network Edge and Node Centrality Variability

Edge weights and centrality indices variability were assessed by using 1000 iterations to bootstrap 95% confidence intervals (CIs) of edge weights [31]. Wide confidence intervals would entangle the interpretation of the edge strength, yet not the presence, since model selection is already performed by LASSO. 

For assessing the variability of the centrality indices (CS-coefficient), participant-dropping subset bootstrap was used [31]. This approach drops a percentage of participants, re-estimates the network, and relates three centrality indices. The CS-coefficient (correlation stability) reflects the maximum proportion of data that can be dropped (ideally >0.25 [11,31]) to retain a correlation > 0.7 with the original centrality indices (95% certainty) [11]. 

## 3. Results

**Figure 1:**
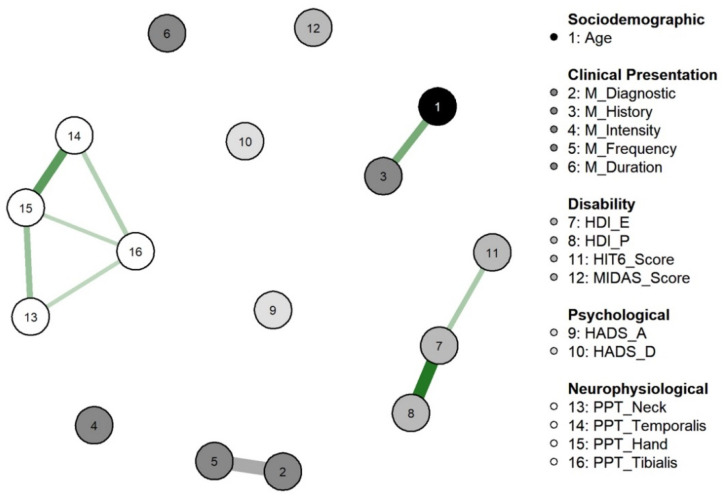
Network analysis of the association between demographic, migraine-related, psychological, and psycho–physical/neuro-physiological measures. Edges represent connections between two nodes and adjusted for the remaining nodes. Direction of the partial correlations were expressed as the color green for positive associations. The color Grey represents the association for categorical variables which no sign is defined. The thickness and color saturation of an edge denotes its weight (the strength of the association between two nodes).

Table 1 summarizes the descriptive data of the sample before and after missing value imputation. The network identified in our sample of 74 women with migraine is showed in Figure 1. Up to 18 correlations were seen between and within the variables. The network identified multiple positive correlations between PPTs among the different locations (nodes 13 to 16), with correlations (ρ) ranging from 0.165 (C5-C6 and tibialis anterior) to 0.409 (hand and temporalis). The strongest associations in the network were between migraine attack frequency and diagnosis of chronic migraine (ρ = 0.634) and between the HDI-E and HDI-P (ρ = 0.545). The rest of correlations ranged from 0.171 to 0.409 (Figure 1). 

Edge weights variability is shown in Figure S1. The non-overlap of the 95% CI of the edge between PPTs at the neck and tibialis anterior locations (nodes 13 and 16) with the 95% CI of the edge between physical and emotional burden because of headache disability (nodes 7 and 8) indicates that the strength of former is significantly greater than the latter.

**Figure 2:**
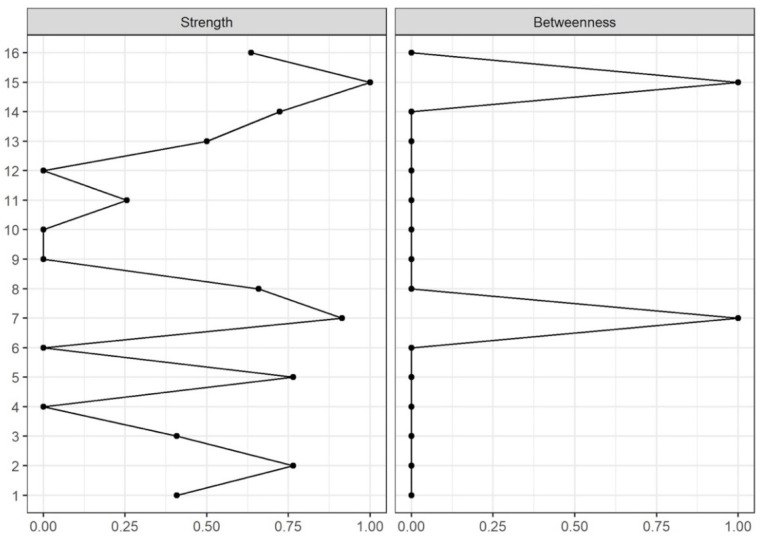
Centrality values (1: maximal importance; 0: no importance) of Strength and Betweenness of each node in the network.

As shown in Figure 2, the node with the highest strength and betweenness centrality was PPT at the second metacarpal (hand), followed by emotional burden because of migraine (HDI-E). 

**Figure 3:**
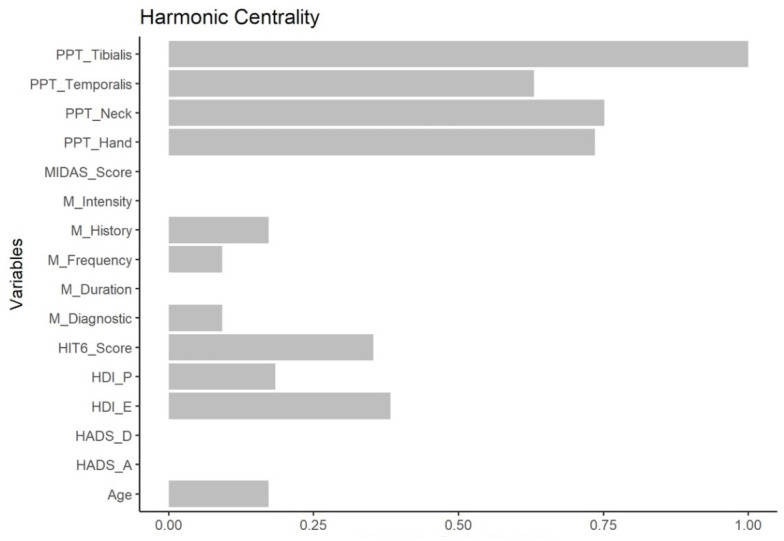
Harmonic Centrality measure (1: maximal importance; 0: no importance) of each node in the network.

The node with the highest harmonic centrality was PPT at the tibialis anterior, followed by PPT at the cervical spine (Figure 3). 

**Figure 4:**
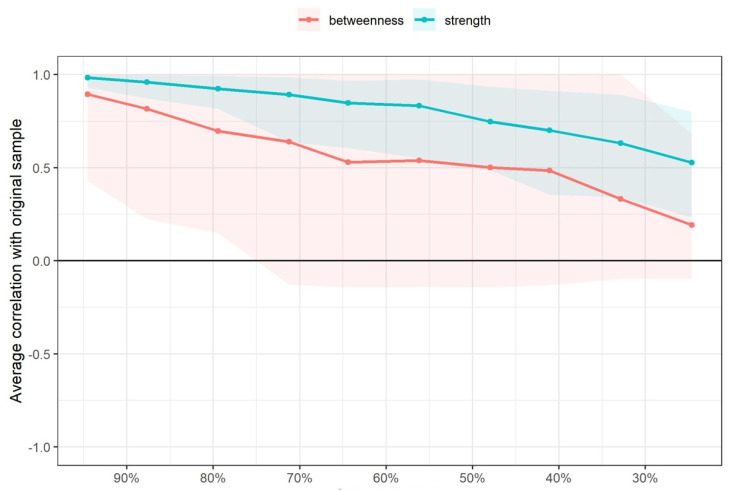
Average correlations between centrality indices of networks sampled with persons dropped and networks built on the entire input dataset, at all follow-up time points. Lines indicate the means and areas indicate the range from the 2.5th quantile to the 97.5th quantile.

The betweenness and strength measures of the network were extremely unstable at CS_cor=0.7_ = 0.000 and CS_cor=0.7_ = 0.287, respectively. The closeness centrality measure could not be assessed with bootstrapping since the resulting networks were unconnected (Figure 4).

## 4. Discussion

Current evidence supports the influence of multiple biopsychosocial mechanisms contributing to the pathogenesis of migraine [4]. This study used a network analysis for better understanding the multivariate interactions between migraine-related, emotional/psychological, or psychophysical variables in women with migraine. The identified network revealed a model where each subgroup of variables (e.g., migraine-related, psychological, and psycho–physical) showed no interaction between each variable.

The identified network identified different clusters grouping each subtype of variables, but without association between them. These results support the multidimensional complexity of migraine. For instance, psycho–physical variables, i.e., widespread PPTs, were grouped in the same cluster. The topic of pressure hyperalgesia in migraine sufferers has been extensively discussed in headache literature [5]. In fact, PPTs in distant pain-free areas, i.e., the second metacarpal and tibialis anterior, exhibited the strongest centrality values. In such a scenario, current results suggest that if clinicians want to influence other migraine-related variables, e.g., migraine pain or disability, included in the identified network, the best variable to focus treatment on would be PPTs, highlighting the relevance of widespread pressure pain hypersensitivity, as a manifestation of sensitization processes in migraine sufferers. This hypothesis would support current assumptions for applying therapeutic interventions such as aerobic exercise or pain neuroscience education for the treatment of migraine [36,37], since these interventions induce centrally mediated hypoalgesia effects. Accordingly, management of the trigeminal area would be also needed to reduce sensitivity of the trigeminocervical nucleus caudalis, the structure responsible for the triggering of migraine attacks and the sensitization process of the nervous system. Further, it should be noted that the network did not identify significant associations of widespread pressure hypersensitivity with migraine clinical parameters. In agreement with these results, previous data suggested no associations between PPTs and clinical outcomes, such as pain and related disability in chronic spinal pain [38]. It is plausible that pressure pain sensitivity reflects the neurophysiological mechanism, whereas headache clinical variables represent a clinical manifestation of pain pathways, hence representing two different aspects of nociception. Nevertheless, it has been found that higher pressure pain sensitivity as expressed by lower PPTs predict future pain and disability in musculoskeletal pain [39]; accordingly, early management of individuals with migraine could help to reduce the possibility of developing higher degree of sensitization. 

The network identified that psychological and migraine-related disability variables did not exhibit significant associations with other variables. A recent meta-analysis has found that depressive and anxiety disorders are the most frequently addressed comorbidities in individuals with migraine and that their prevalence rates seem to be higher than expected [40]. The presence of emotional/psychological disorders support why interventions such as cognitive behavioural therapy have been found to be effective for the management of migraine [41,42]. In fact, psychological interventions are not effective just for improving emotional/cognitive outcomes, but are also effective for reducing headache frequency [43]. In fact, migraine frequency was an independent variable in the identified network, suggesting that it could represent a relevant variable to be addressed in clinical practice. Headache frequency is used to classify patients on episodic or chronic migraine [16,17]. Our sample mostly included women with episodic migraine (75%). The fact that migraine frequency and, accordingly, migraine diagnosis, were independent variables in our network would suggest that identified associations should be similar in either group, episodic or chronic migraine; however, we were unable to conduct separate analyses by group because of the sample size. Future studies should investigate if the identified network is similar (or different) between women with episodic or chronic migraine. 

Similarly, management of migraine-related disability should be the focus of specific therapeutic approaches. In such a scenario, [44] it should be observed that manual therapy interventions could be effective for reducing migraine-related disability as assessed with the HIT-6 scale at short and mid-term, although the level of evidence was very low. In fact, specific manual therapy, e.g., spinal manipulation, has been found to be effective for the reduction in migraine days and pain intensity [45]. The results from this network further reinforce the proposal of applying multimodal therapeutic approaches targeting migraine-related pain and disability (i.e., manual therapy), psychological aspects (i.e., cognitive behavior, and relaxation approaches), health-related (i.e., exercise programs), and also nociceptive mechanisms (i.e., neuroscience pain education programs) [46].

Despite the positive aspects of using a network approach in people with migraine, some limitations are also present. First, the edge weights identified in the network cannot be a source of confirmatory causal inference but may provide indicative potential causal pathways [11]. In other words, biological plausibility between the connected variables is needed from a clinical point of view to determine the viability of the analysis. The current network did not report local associations between the subgroups of variables. Second, we recruited women with migraine from a single university-based headache center; therefore, it may be not representative of general population of migraine sufferers. Additionally, since all individuals were women, accordingly, extrapolation to men with migraine would be not appropriate.

## 5. Conclusions

The application of network analysis in a sample of women with migraine revealed that a model where each subgroup of variables, i.e., migraine-related, psychological, and psycho–physical, showed no interaction between each variable. The network showed that PPTs exhibited the highest centrality measures, supporting a relevant role of pressure pain sensitivity and sensitization in the model. The complex interactions identified in the current network support the contention that migraine treatment should include multimodal therapeutic approaches targeting all these aspects.

## Figures and Tables

**Table 1:** Values (mean ± standard deviation= of baseline characteristics of the total sample (*n* = 74).

Variable	Baseline Scores	Missing Values (*n*; %)
Age (years)	42.3 ± 12.1	0; 0
Migraine Type (*n*; %)		
Episodic (1)	56; 75.6	0; 0
Chronic (2)	18; 24.4	0; 0
Years with Migraine (years)	19.6 ± 13.9	0; 0
Migraine Intensity (0–10)	8.1 ± 2.0	0; 0
Migraine Frequency (*n*/month)	9.9 ± 8.1	0; 0
Migraine Duration (hours/episode)	24.2 ± 20.6	0; 0
HDI-E (0–52)	27.0 ± 13.4	0; 0
HDI-P (0–48)	34.7 ± 11.5	0; 0
HADS-A (0–21)	12.3 ± 2.5	0; 0
HADS-D (0–21)	10.5 ± 3.0	0; 0
HIT6 (36–78)	63.0 ± 7.3	0; 0
MIDAS (days missed work)	46.3 ± 69.3	1; 1.35
PPT Neck (kPa)	135.4 ± 46.5	0; 0
PPT Temporalis (kPa)	156.7 ± 61.6	0; 0
PPT Hand (kPa)	194.5 ± 64.1	0; 0
PPT Tibialis Anterior (kPa)	327.0 ± 114.2	0; 0

HADS: Hospital Anxiety and Depression Scale; HDI: Headache Disability Index; HIT: Headache Impact Test; MIDAS: Migraine Disability Assessment Scale; PPT: Pressure Pain Thresholds.

## Data Availability

All data derived from this study are presented in the text.

## Supplementary Materials

The following supporting information can be downloaded at: https://www.mdpi.com/article/10.3390/diagnostics12102318/s1, Figure S1: Bootstrapped 95% quantile confidence interval of the estimated edge weights of the network. “Bootstrap mean” reflects the average magnitude of edge weights across the bootstrapped samples. “Sample” reflects the magnitude of edge weights of the original network built on the entire input dataset.
