# Supplementary figures and images for: Network Analysis Reveals That Headache-Related, Psychological and Psycho–Physical Outcomes Represent Different Aspects in Women with Migraine
Source: Diagnostics (Basel). 2022 Sep 26;12(10):2318. doi: 10.3390/diagnostics12102318 (PMC9600561; doi:10.3390/diagnostics12102318)

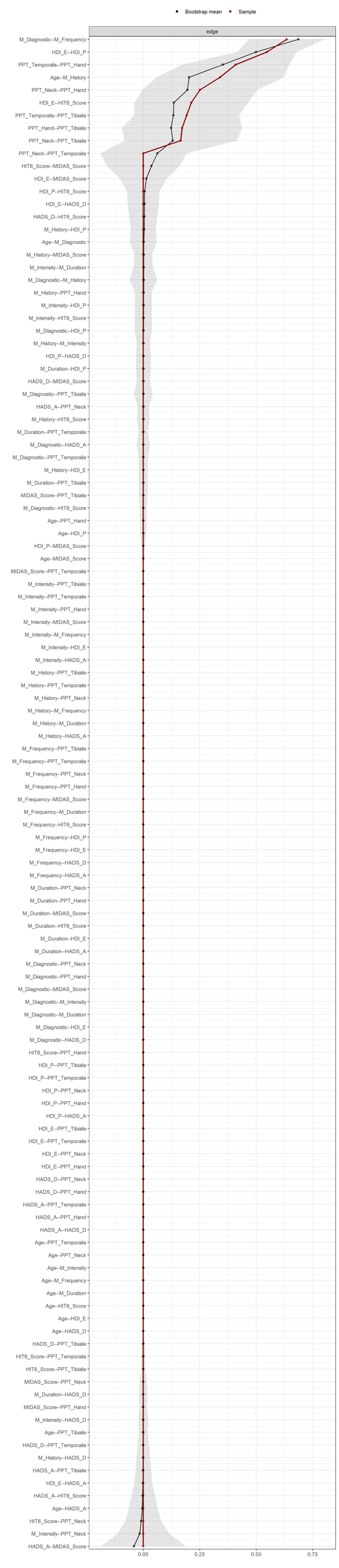

Supplement: Supplementary file 1 [file diagnostics-12-02318-s001.zip › Supplementary Figure S1.png]
